# Determinants of Implementing an Information and Communication Technology Tool for Social Interaction Among Older People: Qualitative Content Analysis of Social Services Personnel Perspectives

**DOI:** 10.2196/43999

**Published:** 2024-02-26

**Authors:** Johanna Fritz, Petra von Heideken Wågert, Annelie K Gusdal, Rose-Marie Johansson-Pajala, Caroline Eklund

**Affiliations:** 1 School of Health, Care and Social Welfare Mälardalen University Eskilstuna/Västerås Sweden

**Keywords:** information and communication technology, implementation, determinants, social isolation, loneliness, organization, digitalization, facilitators, barriers, older people

## Abstract

**Background:**

Older people are particularly vulnerable to social isolation and loneliness, which can lead to ill-health, both mentally and physically. Information and communication technology (ICT) can supplement health and social care and improve health among the vulnerable, older adult population. When ICT is used specifically for communication with others, it is associated with reduced loneliness in older populations. Research is sparse on how the implementation of ICT, used specifically for communication among older people in social services, can be performed. It is recommended to consider the determinants of implementation, that is, barriers to and facilitators of implementation. Determinants related to older people using ICT tools are reported in several studies. To the best of our knowledge, studies investigating the determinants related to the social services perspective are lacking.

**Objective:**

This study aims to explore the determinants of implementing the Fik@ room, a new, co-designed, and research-based ICT tool for social interaction among older people, from a social services personnel perspective.

**Methods:**

This study used an exploratory, qualitative design. An ICT tool called the Fik@ room was tested in an intervention study conducted in 2021 in 2 medium-sized municipalities in Sweden. Informants in this study were municipal social services personnel with experience of implementing this specific ICT tool in social services. We conducted a participatory workshop consisting of 2 parts, with 9 informants divided into 2 groups. We analyzed the data using qualitative content analysis with an inductive approach.

**Results:**

The results included 7 categories of determinants for implementing the ICT tool. *Being able to introduce the ICT tool in an appropriate manner* concerns the personnel’s options for introducing and supporting the ICT tool, including their competencies in using digital equipment*. Organizational structure* concerns a structure for communication within the organization. *Leadership* concerns engagement and enthusiasm as driving forces for implementation. *The digital maturity of the social services personnel* concerns the personnel’s skills and attitudes toward using digital equipment. *Resources* concern time and money. *IT support* concerns accessibility, and *legal liability* concerns possibilities to fulfill legal responsibilities*.*

**Conclusions:**

The results show that implementation involves an entire organization at varying degrees. Regardless of how much each level within the organization comes into direct contact with the ICT tool, all levels need to be involved to create the necessary conditions for successful implementation. The prerequisites for the implementation of an ICT tool will probably change depending on the digital maturity of future generations. As this study only included 9 informants, the results should be handled with care. The study was performed during the COVID-19 pandemic, which has probably affected the results.

## Introduction

Older people are especially vulnerable to social isolation and loneliness, particularly because they are exposed to risk factors such as living alone and experiencing chronic illness [1]. There is robust evidence linking loneliness and isolation with physical decline; morbidity; increased mortality; and cognitive and mental health problems, such as depression and dementia; and increased risk of suicide [2-7]. Several studies point toward an increase in loneliness during the COVID-19 pandemic with *stay-at-home* orders and recommendations for social distancing [8-11], and the impact is particularly severe among people aged ≥80 years [12]. Information and communication technology (ICT) can supplement health and social care and improve health in the vulnerable, older population [4,13]. ICT is a part of welfare technology, which in one way or another, improves the lives of those who need it. When technology is used specifically for communication with others, it is associated with reduced loneliness [4,13-16] and increased well-being and life satisfaction [4] in older populations. Technology can improve social connectedness among older adults. The specific effectiveness rates favor ICT and videoconferencing [13]. Chen and Schultz [15] identified 4 important mechanisms for reducing social isolation using ICT: staying connected to other people, such as family and friends; gaining social support; participating in interesting activities; and boosting self-confidence. Studies show that ICT can support and maintain the social relationships and healthy and independent lives of older people at the individual level and should be prioritized as an early and preventive intervention in social services [17]. However, the use of ICT has been shown to decrease after 6 months of use [15,16]. Only few studies have investigated how the implementation of web-based social activities in social services can be performed. Thus, there is a need for sustainable, structured, and well-planned solutions for the implementation of ICT in social services.

To make ICT useful for older people, social services must consider the determinants for its implementation, that is, barriers to and facilitators of implementation. Determinants related to older people are reported in several studies. A literature review including 59 papers identified determinants related to this specific population’s adoption of technology, such as perceived usefulness, potential benefits, user friendliness, ease of learning, perceived costs and savings, knowledge about existence, availability in the market, technical support, social support, perceived emotional and psychological benefits, and relevance with their previous experiences [18]. Other identified determinants related to older people are gaps in ICT literacy, fear of making mistakes when learning the ICT tool [19], privacy concerns, technical difficulties, lack of user-friendly options designed specifically for an older population, and lack of experience in using technology [20,21]. However, implementation of ICT does not depend on the older people alone. Social services personnel have an important role in introducing ICT to older people and to support its use [22]. Thus, ways of working to introduce and support the use of ICT among older people need to be implemented in the social services and other services they offer. To the best of our knowledge, determinants of implementing an ICT tool for social interaction among older people related to the social services personnel perspective are lacking.

The readiness and maturity to adopt digitalization and new ways of working vary among the social services provided by Swedish municipalities, for example, in residential care services and home care services. Few older people receive access to welfare technology services despite the benefits [23]. It is a large step for an organization to move from a limited project to implementation in their organization. Konttila et al [24] identified determinants of importance for digitalization in health care but not specifically for the care of older people, related to professionals’ knowledge, skills, attitudes, and experiences and organizational and collegial support. One of the proposals from the Swedish Ministry of Health and Social Affairs [23] is that studies are needed to implement models for welfare technology in social services. A systematic review focused on facilitators and barriers that influence the implementation of welfare technology for older people, from the perspectives of older individuals, people with disabilities, informal caregivers, health and care personnel, organizations, infrastructure, and technology [25]. Overall, 6 themes of determinants were identified: capacity, attitudes and values, health, expectations of effects, shared decision-making, and identity and lifestyle. These determinants are within different levels in an organization and are consistent with other determinant frameworks for implementation [26,27]. However, most of the included papers in the systematic reviews of determinants for digitalization and implementation of welfare technology for older people [24,25] involved various types of technology, such as technology for smart homes, mobile devices in medicine and public health, self-care, medication, and surveillance systems, whereas ICT used specifically for communication among older people was not included. This study aimed to explore the determinants of implementing the Fik@ room, a new, co-designed, and research-based ICT tool for social interaction among older people, from a social services personnel perspective.

## Methods

### Design

This study used an exploratory, qualitative design [28,29]. An exploratory design is appropriate for conducting studies in a field that is relatively underexplored and hence, an inductive approach was adopted [30].

### The Fik@ room: An ICT Tool for Social Interaction Among Older People

**Figure 1:**
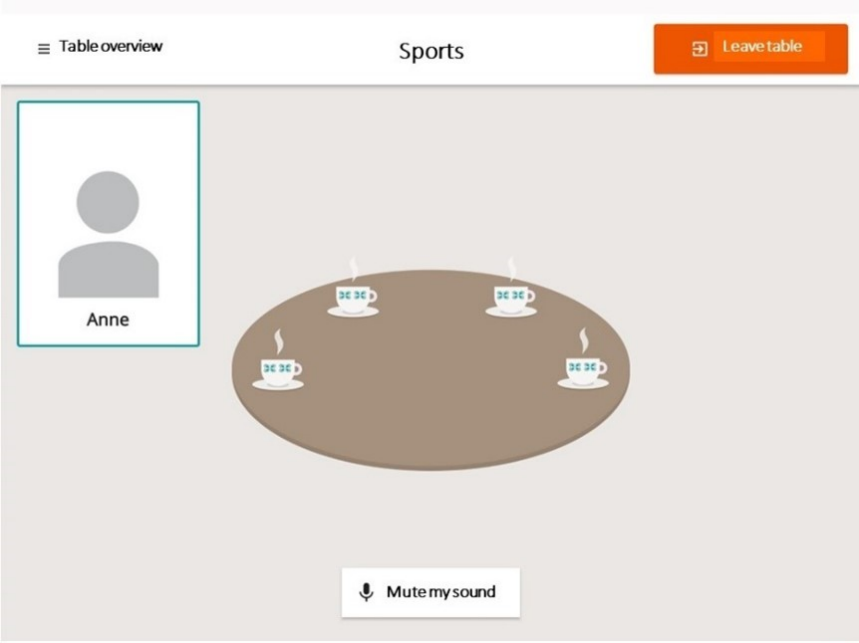
Illustration of a digital coffee table within the Fik@ room. The conversation theme of this table is sports, and in this example, only 1 person is attending.

The Fik@ room is a research-based ICT tool, in the form of a web platform for safe web-based social interaction, created and developed by researchers in coproduction with older people aged ≥60 years, municipal health and social care personnel, and an IT company, based on focus group interviews and workshops. The content and design of the Fik@ room was based on the needs and wishes expressed by older people [31]. The older people who participated in the development process in 2019 and 2020 expressed the importance of knowing that others visiting the Fik@ room were equal, that is, experiencing loneliness, and access to the Fik@ room was protected by an individual password. This log-in procedure contributes to increase the safety and the feeling thereof for the older people. All older people with access to the Fik@ room have received personal log-ins from a contact person from the municipality. People who received log-ins were older people who had experienced loneliness or social isolation [31]. The Fik@ room consists of digital coffee tables with seating for up to 4 people at each table (Figure 1). The older people can start conversations regarding topics of their own choice using video, voice, or chat. They can also post messages to each other on a bulletin board. In the Fik@ room, older people can meet new friends and socialize as a way of forming routines in their everyday lives. The Fik@ room focuses on meeting peers (people in the same situation) and offers the opportunity to meet and discuss subjects related to users’ interests. This foundation improves the quality of conversations in the Fik@ room and increases independence, participation, accessibility, and options for users to form their own social interactions as part of their everyday lives, which is associated with better quality of life for older people [17]. The Fik@ room is available on Google Play Store and Apple App Store (for iPad devices) but will not be available for logging in without permission from an authorized gatekeeper such as personnel in the municipality.

### Informants and Settings

The ICT tool was tested in an intervention study performed in May and June 2021 in 2 medium-sized municipalities in the middle of Sweden. The 2 municipalities were chosen because they are coproduction partners of the University and were both involved in the development of the Fik@ room. Informants in this study were social services personnel, with experiences of the intervention study and thus the implementation of this specific ICT tool in social services: municipal social services managers and social services personnel who recommended the ICT tool to older people in the intervention study (Table 1). The informants were chosen using a purposive sampling technique to capture different perspectives and experiences from the stakeholders in the implementation process, which provided the process with experience-related information and theory-based knowledge. In total, 9 informants from the 2 municipalities agreed to participate, who were basically all the people involved in the implementation process. Municipality 1 had an ongoing digitalization project running in parallel with the intervention study. This means that the municipality was appointed by The Swedish Association of Local Authorities and Regions as 1 of 10 model municipalities, which would function as a model for the digitalization of care for older people. These 10 municipalities received extra financial support to enable time for knowledge dissemination. Together with The Swedish Association of Local Authorities and Regions, they support other municipalities with knowledge about digital services and welfare technology. Municipality 2 was not involved in the parallel, ongoing digitalization project.

**Table 1:** Characteristics of the informants and settings.

Characteristics		Municipality 1 (n=6)	Municipality 2 (n=3)
**Sex of the informants, n (%)**			
	Male	2 (33)	0 (0)
	Female	4 (67)	3 (100)
**Profession of the informants, n (%)**			
	Manager	2 (33)	1 (33)
	Developer	1 (17)	0 (0)
	Occupational therapist	0 (0)	1 (33)
	Technology supporter^a^	2 (33)	0 (0)
	Guide for older people^b^	1 (17)	1 (33)
Ongoing digitalization project		Yes	No

^a^Technology supporters were IT experts, employed in the municipality, who prepared the iPad devices for the older people in the intervention study by installing SIM cards and connecting them to the network and installing the Fik@ room app.

^b^Guides for older people were social services personnel with experience in IT, employed in the municipality, who worked at the meeting places for older people and supported the older individuals regarding the use of the iPad and the Fik@ room app.

### Data Collection

Participatory workshops with 2 researchers acting as workshop leaders provided a valuable opportunity to learn together and discuss several perspectives. The informants both generated and analyzed data through a structured process for data collection and analysis that includes a combination of individual and group activities, inspired by the effect modifier assessment (EMA) method [32]. The EMA method consists of workshops and subsequent analyses. The workshop leaders facilitate the collection of information on past significant events; in this study, the event in focus was the introduction of the Fik@ room to older people. The method facilitates a combination of individual and group activities, which implies that all experiences from each informant is used and then developed in a group discussion. In this study, semiquantitative estimations were not used because all the determinants (barriers and facilitators) were considered important regardless of how many people had experienced them. The workshop guide (Multimedia Appendix 1) could be considered as a semistructured interview guide, but the workshop informants interactively influenced the interview guide by deciding what events and in what way the events are discussed. Each informant participated in 1 or 2 participatory workshops in August and September 2021, in groups of 3 to 6 participants. Each workshop began with casual conversation to help the informants feel at ease and more comfortable in the setting. The researchers served as workshop leaders to encourage a flow of discussion. To create a comfortable environment for the informants, the workshops were conducted separately for each municipality, ensuring that all the informants within a workshop were familiar with one another. The participatory workshop consisted of 2 parts. The first part focused on the determinants for implementing the ICT tool, and the workshop activities involved the identification of possibilities, obstacles, and challenges that were experienced during the intervention study. The first workshop leader (JF) asked the informants to individually note the possibilities, obstacles, and challenges on a paper in front of them and, thereafter, facilitated a group discussion regarding the same questions. The second workshop leader (CE) asked clarifying questions during the workshop and summarized the discussion at the end of the workshop. The second part started with a review of the first part, followed by the same individual and group processes as the first part, focusing on scenarios for the development of methods to support the implementation of the ICT tool. The workshop activities were regarding how to overcome the obstacles and reinforce the possibilities identified in the first part of the participatory workshop.

The workshops were performed using a web-based video communication tool, Microsoft Teams (Microsoft Corp). Municipality 1 performed the 2 parts of the workshop in separate sessions (2 hours each), and municipality 2 performed both the parts in the same session (2 hours). The informants’ professions were requested at the beginning of the first workshop. The workshops were video recorded using the Microsoft Teams video platform.

### Data Analysis

The data were analyzed using qualitative content analysis with an inductive approach [33]. The recordings were transcribed verbatim. The text was read several times for familiarization. Meaning units related to the determinants for implementing the ICT tool for social interaction among older people from the perspectives of the social services personnel were identified, coded, and grouped into subcategories and categories according to similarities. Examples of the abstraction of categories from meaning units are shown in Table 2. The identification of meaning units and categorization were performed by the first author. To validate the analysis, part of the categorization was also performed by 2 other authors (CE and PvHW) separately. In addition, the analysis was regularly discussed and validated among all the authors during the process to achieve consensus.

**Table 2:** Examples of the abstraction process: meaning units, codes, subcategories, and categories.

Meaning unit	Code	Subcategory	Category
...That they [the personnel] have a login so when they are there, they [personnel and older person] can talk to each other at a table [within the ICT^a^ tool] just like we did.	Be able to log in to the ICT tool	Be able to show the tool	Be able to introduce the ICT tool in an appropriate manner
If it had been as usual [before the pandemic] then we would have done the same at home visits, and it would have been much easier because then you can show this leaflet, talk about it, and sell it in a better way, so it would have been much better.	Easy to show in person	Be able to show the tool	Be able to introduce the ICT tool in an appropriate manner
The pandemic, of course, because it has not been possible to visit people. They have not wanted to let us in, and not even homecare staff have been able to visit some people. They have declined home care and arranged help in another way because they do not want to expose themselves to the coronavirus.	The pandemic hindered in-person visits	Be able to show the tool	Be able to introduce the ICT tool in an appropriate manner

^a^ICT: information and communication technology.

### Ethical Considerations

This study was conducted in accordance with the Declaration of Helsinki [34] and Swedish Ethical Review Act [35]. However, according to the act, ethics approval by the Swedish Ethical Review Authority is not needed when, for example, sensitive personal data are not collected (ie, when interviewing staff to determine how they perform their work), as in this study. However, the intervention study (in which older people were study participants) has obtained ethics approval by the Swedish Ethical Review Authority (Dno 2020-06640). Participation was voluntary, and all informants provided their verbal consent after receiving verbal and written information. Furthermore, they were allowed to withdraw at any time without consequences.

## Results

### Overview

The results included 7 categories of determinants for implementing the ICT tool: be able to introduce the ICT tool in an appropriate manner, organizational structure, leadership, digital maturity of the social services personnel, resources, IT support, and legal liability (Textbox 1). The results are presented using the categories as headings, and the subcategories are italicized in the text.

**Categories and subcategories of determinants for implementing the information and communication technology (ICT) tool for social interaction among older people.**

**Be able to introduce the ICT tool in an appropriate manner**

- To tailor the information
- Be able to show the ICT tool
- To let the user practice
- The personnel need to have knowledge about the ICT tool

**Organizational structure**

- A system for effective communication
- Clear roles

**Leadership**

- The manager’s engagement
- Enthusiasts

**The digital maturity of the social services personnel**

- The personnel’s digital skills
- The attitudes among the personnel varied

**Resources**

- Costs
- Time
- Access to transport

**IT support**

- Accessibility
- Follow-up system

Legal liability (no subcategories)

### Be Able to Introduce the ICT Tool in an Appropriate Manner

Older people need to be informed about the existence of the ICT tool. Reaching all potential users with information about the tool was a challenge that the informants did not know how to address. They found that written information sent via mail reached many potential users and was easy to distribute. However, when introducing the ICT tool to an older person, the informants found it important *to tailor the information*, for example, the amount of information that the older person was able to receive, according to their knowledge and attitude toward ICT. Knowing the person and meeting in person facilitated tailoring compared with written information and web-based meetings:

I have to know how to structure the conversation with the person I am calling, and I also have to do that when I call the person because I have to hear what status the person has, how should I handle the person, i.e., how should I structure my conversation so that I establish good communication with the person. I must choose my conversational tone mode, how I present it, and how I tell it, a lot is about structuring the conversation and I have to do that immediately when I get in touch with the person.

Informant 4

When introducing the ICT tool, the informants found it important to *be able to show the tool*. To enable this, the personnel also needed to have the prerequisites to log in to the ICT tool, which was not always a matter of course. The personnel also needed to have access to their own account, and the program needed to be installed in their digital equipment. To reduce older people’s fear of digital tools, the informants found it important *to let the users practice* using digital tools in a playful manner. Getting acquainted with digital equipment, such as tablets or computers, through playing games, watching movies, or reading newspapers can improve the ease of use of other digital tools such as the ICT tool. Some meeting places for older people offered these practices and integrated digital tools into their daily services. During the COVID-19 pandemic, in-person visits were not always possible but were considered a prerequisite for showing and practicing the ICT tool:

To dare to use the tablet...to use it for something they are familiar with, read the newspaper or whatever it may be, as a first step. It may not be the ICT tool that is the first step, but it may be the next step when they have learned to use the tablet. A game can be a little easier or reading the newspaper can be an easier way to start using it and then you take the next step.

Informant 7

To be able to provide information regarding the ICT tool, *personnel need to have knowledge about it*. The informants perceived the written information regarding the ICT tool as useful for understanding the purpose of the tool. However, it was difficult to inform older people about a tool without knowing how it looked or how it worked. Having both seen and tried the tool made the personnel more confident when informing older people about it:

It was great that we got to try the ICT tool first, because it also makes it easier when you are out with the user to show them how it works because you actually know what the picture looks like, how the sound sounds, what happens with the sound if we sit too close to each other. So, I thought it was great, you need to try it first.

Informant 6

### Organizational Structure

When implementing the ICT tool, the informants perceived it as important to have *a system for effective communication* within the organization. A secure electronic communication system regarding personal data worked appropriately for communication among different parts of the organization regarding lists of potential users, who to call, who would visit whom, information needed for home visits, and so on. After the personnel had visited an older person, they used the same system for feedback about the visit and to document whether further support was needed. The informants also highlighted the importance of feedback among different levels within the organization, such as to the management team by whom new decisions could be made:

Lists were created so that we had a structure on which we had called, who would go to whom, all the information needed to make a home visit and even be able to write when you had been there, and it was a completed project...So, it was a very good structure in the lists.

Informant 3

Spontaneously, I would say that some structure will be required for the recruitment of these [older] people.

Informant 2

The informants expressed that *clear roles* facilitated the implementation of the ICT tool. Clarity about whom to ask regarding a special issue, whom to ask for support, and so on makes the work more efficient. Similarly, the person who holds the role knows what duties come with the role. They experienced that the implementation was facilitated if the selected personnel performed the introduction, skills training, and support of the ICT tool:

I thought a bit about this regarding whom to contact and so on. It should be incredibly clear, both for our users but for the employees as well. They should not have to think “who are we to contact to get support for this?” but there must be somewhere very, very clear so it can be done quickly, so that you do not have to run around and look and waste time looking for who to contact.

Informant 7

### Leadership

The informants expressed that *the manager’s engagement* was important to the implementation, and it became obvious when engagement was lacking. A manager who was open to the ICT tool and interested in its implementation spread their engagement to the rest of the working group. According to the participants, another success factor was having *enthusiasts* or champions as leaders. These were selected personnel with a clear mandate within the organization who had extra knowledge about ICT, who worked actively regarding its implementation, and who regulated the entire process:

That it is someone who owns the question, who has the question on their table, who is the one who then ensures that it is followed up, and the continuity of the whole thing I think is very important.

Informant 6

### The Digital Maturity of the Social Services Personnel

The implementation of the ICT tool was affected by *the social services personnel’s digital skills*. According to the informants, some of the personnel did not know how digital equipment worked, such as a tablet, which hindered them from informing others about and supporting the use of the ICT tool:

The personnel couldn’t use a tablet either, you had to give them basic instructions on how to press the button to start it up. Of course, there were also those who were very talented. But you might think that it is only the older people that don’t have the skills, but it is actually the personnel too.

Informant 3

The informants expressed that *the attitudes among the social services personnel varied*. Some were very interested, and the implementation was conducted smoothly. In other parts of the organization, the personnel did not even talk about the ICT tool. The informants thought that an ICT tool that can be used by both older people and personnel would create great interest among the personnel compared with a tool that can be used only by older people. For example, the personnel could conduct lectures or discussions about health-related subjects, such as diet and exercise, within the ICT tool. The informants thought that this digitalization investment had a positive impact on personnel’s attitudes toward digital tools, which provided synergies and paved the way for the implementation of this specific ICT tool:

Yes, but what if we can have a table where we can talk about health and diet, and those who want to can come in and hear, listen, or participate in discussions.

Informant 2

### Resources

Implementation requires resources of various types. In this case, the informants highlighted resources regarding *costs*, *time*, and *access to transportation*. They expressed concern about the *costs* that would be required to gain access to the tool and support. The implementation of the ICT tool would be at the expense of something else. According to the informants, a payment model based on different fees, depending on what is included, may facilitate implementation:

If you start from the scenario that it is the municipalities that in some way buy a license or something similar for the ICT tool, then the payment model itself could be for a lower amount if the municipality itself, so to speak, moderates what is said and not, and perhaps a higher amount then if it is a company who would be responsible for it.

Informant 5

Implementation takes *time,* and some of the most time-consuming parts, as mentioned by the informants, were sending and following up on information letters, delivering tablets, creating log-ins, and showing users what to do. The informants felt that time had been allocated at different levels (eg, to key individuals within the digitalization investment area of the model municipality):

It does not matter what we are going to do, time is required. And if you have decided to make a change like this, you should be aware that time is needed. So, it is obvious that it is something that really needs to be considered if it would be implemented somewhere else as well. That you actually make sure you have that time and resources, it costs to implement something, but in the end, it can generate so much more.

Informant 7

*Access to transportation* is a prerequisite for home visits. The informants experienced that there are always cars available as a means of transportation for home visits.

### IT Support

The *accessibility* of the support, both for the users and personnel, was perceived as important for implementing the ICT tool. Contact information needed to be available, and it was preferable if all support could be reached using the same contact method (eg, the same phone number or email address). In addition, time needs to be allocated for support. According to the informants, support not only involved direct contact with the user but also involved communication with and recurring feedback between the supporters and the personnel. Support could be provided through various forums to increase accessibility, such as during home visits, at meeting places for older people, or at the public library. Support could also be provided via other digital media, which hindered accessibility, as the requested support was sometimes related to the difficulties in handling the digital equipment itself and, thus, also the digital support. During the COVID-19 pandemic, the accessibility of support was particularly limited, as digital support could not be received owing to some users’ lack of skills, and the older people did not accept home visits owing to the risk of spreading the infection:

What needs to be strengthened is time, it is the key that we have talked about. It was also what was difficult, but we also saw that what still worked well was when there was allocated time for various steps: time to be at home with the user to practice and provide support and follow-ups, but also time for communication with personnel and recurring feedback: to ask how it works out. So, I think that resources and time really need to be strengthened, then you have all the prerequisites to succeed.

Informant 6

The personnel lacked a *follow-up system* for support. They expressed a need to be able to follow up regarding whether the user had used the ICT tool to facilitate implementation. It was not possible to assess whether the older person understood how to use the ICT tool after a short introduction. The informants suggested that it should be possible to obtain information about the number of log-ins on the ICT tool to be able to follow up with users who have few log-ins:

It would be interesting to get feedback on if these users have not been in at all. Then you could have maybe called them and asked: How are you? Do you want more help?

Informant 9

### Legal Liability

The informants expressed doubts about whether the municipalities complied with the legislation if the ICT tool was offered by the municipality and used inappropriately, such as the use of racist statements. There was a concern that the municipality cannot guarantee that nothing inappropriate is said within the ICT tool without some form of supervisory function. Therefore, it was proposed that a moderator of the ICT tool could perform that function:

If the municipality buys it, we stand as some form of guarantor, we also have a responsibility not to release that freely, but to have some form of moderating function that can support what is said. Because racist statements may be used, for example, and then we also have a responsibility to take care of it.

Informant 5

## Discussion

### Principal Findings

The results included 7 categories of determinants for implementing the ICT tool. *Being able to introduce the* ICT *tool in an appropriate manner* concerns the personnel’s options for introducing and supporting the ICT tool, including their competencies in using digital equipment*. Organizational structure* concerns a structure for communication within the organization. *Leadership* concerns engagement and enthusiasm as driving forces for implementation. *The digital maturity of the* *social services personnel* concerns the personnel’s skills and attitudes toward using digital equipment. *Resources* concern time and money. *IT support* concerns accessibility, and *legal liability* concerns possibilities to fulfill legal responsibilities*.*

### Comparison With Previous Studies

Determinants of implementing ICT tools related to older people have been reported in several studies and systematic reviews [18-21]. The novelty of our study lies in its knowledge about the determinants of implementing a new, co-designed, and research-based web platform, customized specifically for older adults, from a social services personnel perspective. The determinants identified in this study are concretized to increase the understanding of specific factors that influence the implementation of an ICT tool for social interaction among older people. Damschroder et al [27] and Flottorp et al [26] highlighted several domains of determinants, including factors related to the implemented intervention itself (in this case, the ICT tool) and patient factors. These 2 domains are not presented in our results but are reported in a related article [22]. The social services personnel’s view about the determinants identified in this study were mainly related to the interaction between the personnel and older individual, and organizational factors. Our findings differ from those of other studies describing older people’s views about determinants, which were mainly related to their own capacity, attitudes, and health-related benefits and the usefulness and ease of learning the technical tool [18-22]. However, the interaction between the professional and the older individual relates to strategies for supporting older people to overcome barriers mentioned as determinants by the older people themselves. Thus, although the determinants mentioned by social services personnel and older people differ, they are logically interconnected. The 7 categories of determinants reported in this study correspond to the following domains reported in the paper by Flottorp et al [26]: individual health professional factors; professional interactions; incentives and resources; capacity for organizational change; and social, political, and legal factors. Some of the factors within these domains were not mentioned as determinants by the informants in our study, such as the continuing education system, assistance (external) for organizational change, contracts, and political stability. As they were not mentioned by the informants, we interpreted them to be of less importance in this specific case.

Most of the identified determinants in our study were related to contextual factors at different levels, which confirms that contextual determinants play an important role in implementation [36]. Nilsen and Bernhardsson [36] highlighted contextual factors as determinants at the micro (interaction between the professional and patient), meso (the organization), and macro (influences from the wide environment) levels. The micro-meso-macro framework for analysis is a useful way of understanding the determinants of implementation, as implementation is a multilevel phenomenon [37]. In this study, the contextual determinants mostly involved the micro (*be able to introduce the ICT tool in an appropriate manner*) and meso levels (*organizational structure, leadership, resources*, and *IT support*). Only 1 category was identified at the macro level (*legal liability*). The Lancet and Financial Times Commission on Governing Health Futures 2030 [38] recommends interventions at the macro level to facilitate the digitalization of health and social care to achieve future health and well-being. From the perspective of the personnel, the impact of the identified determinants likely differs. As macrolevel determinants were not mentioned by the informants to the same extent as microlevel and mesolevel determinants, it could be assumed that macrolevel determinants were not perceived to have as great an impact as micro- and mesolevel determinants on the implementation of the ICT tool.

A category of determinants that we identified, *the digital maturity of the social services personnel,* was related to individual health professional factors according to the checklist by Flottorp et al [26] and, in particular, knowledge, skills, and cognition. A lack of digital competence has been identified across all professions within social services in Sweden, and the development of the personnel’s competence is stated to be a success factor when implementing welfare technology in social services [23]. Konttila et al [24] recommend that learning how to use technical devices should be integrated into the personnel’s daily work by providing education and sufficient time for learning. In previous studies, knowledge and skills primarily focused on digital and technical aspects. However, our results in the category, *be able to introduce the ICT tool in an appropriate manner*, also emphasize the importance of the personnel’s pedagogical knowledge and skills in teaching older people how to use an ICT tool. This introduces additional demands on the personnel that must be considered when implementing an ICT tool in social services.

Our results are consistent with the barriers to and facilitators of the implementation of welfare technology identified by Zander et al [25]. All our identified determinants correspond to the themes reported by Zander et al [25] regarding capacity, attitudes, and values. In addition, we identified enthusiasts, as part of the *leadership* category, as an important driving force for implementation, which was not explicitly mentioned by Zander et al [25]. However, similarity can be seen with the theme of participation, as Zander et al [25] discussed the importance of involvement in the development, decision-making, and implementation processes as a facilitator of implementation. A theme of determinants that Zander et al [25] identified but were not identified in our results was expectations. Expectations were seen as a barrier to the implementation of welfare technology and were related to fear that the technology would affect the quality of care, threats to professional identity, and fear of losing jobs. It is possible that the ICT tool in our study was not perceived as a threat against the personnel’s professional role, as it did not directly affect the quality of care or replace the care provider. It is also important to remember that the technology used in the literature review by Zander et al [25] did not include ICT used specifically for communication among older people, which can explain the differences in the results.

According to our results, enthusiasts seemed to be an important facilitating determinant for implementation. Enthusiasts can also be described as champions or local opinion leaders, depending on whether they are appointed by the management or considered informal, educationally influential leaders appointed by peers [39]. In our results, we interpret enthusiasts more consistently with the definition of champions. However, it remains unclear whether the enthusiasts only function through managerial status and process or also function through social influence, such as an opinion leader. To support implementation, the evidence for the role of local opinion leaders is more robust than that for champions, and it seems that involvement of local opinion leaders is an effective implementation strategy [39,40]. To understand the impact mechanisms of enthusiasts, the role and significance of enthusiasts need further clarification.

One of the 2 municipalities included was a model municipality for the digitalization of care for older people, which contributed to an important difference between these municipalities. The informants from the model municipality expressed the determinants by describing their own experiences as facilitators (eg, their experiences regarding how a system for effective communication among personnel facilitated the implementation of the ICT tool). The other municipality talked about the same determinant as a barrier, that is, the lack of a system for effective communication. It was obvious that the digitalization project positively influenced the implementation of the ICT tool, which also confirms the importance of determinants related to the organization. Although the ICT tool was supposed to be easy to use for older people [31], it is still important to have an organizational structure to support the older people in using the ICT tool. Previous studies highlight the importance of a shared vision within the organization for the implementation and involvement of leadership [25]. These determinants were perceived by the informants in the model municipality. They also experienced other facilitators identified in previous studies, such as a system for communication, clearly defined roles, enthusiasts, access to IT support, and resources that facilitated the implementation [24,25]. Digital maturity appears to be great in the model municipality, which also affected the implementation of the ICT tool.

Implementation is more likely to be successful if implementation strategies are chosen based on an assessment of determinants (facilitators and barriers) of implementation [40]. However, when selecting the implementation strategies, consideration must also be given to the effectiveness of different strategies based on well-designed studies and systematic reviews, the phases—implementation or maintenance—of the actual implementation process [41], and the theoretical underpinnings of the implementation [42,43]. Our findings can contribute to increasing the understanding of the complexity of implementing an ICT tool for social interaction among older people in municipal settings and guide the choice of implementation strategies.

Several studies have explored the determinants of using ICT tools, often in relation to older people. ICT tools can mean different types of technology that are used in health and social care and used by older people outside health and social care. Most ICT tools studied are not designed specifically for older people [18-21,23-25]. In this study, determinants are explored in relation to the Fik@ room, a specific ICT tool for social interaction among older people, developed for and in coproduction with older people. As ICT tools involve large variety, it can hinder the transferability of our results. Therefore, it is important that the readers themselves are aware of the type of ICT tools that are studied.

### Strengths and Limitations

As determinants for improving professional practice have been identified at different levels [26,41], we wanted to include the informants involved in the implementation of the ICT tool in different ways. The combination of informants working with leadership and informants working directly with the older people in different ways enabled a comprehensive understanding of the determinants of the implementation of an ICT tool for social interaction among older people in municipal settings. It would have been desirable to have more informants from municipality 2. Although the study only included 9 informants, these informants had specific experiences pertinent to the study’s aim by being involved in the intervention study that introduced the Fik@ room to older people. Taken together with the specific phenomenon in question and a narrowly defined objective, the data were considered to have sufficient information power [44].

The use of participatory workshops inspired by the EMA method [32] contributed to a systematic approach to data collection and opportunities to learn from each other. The combination of individual and group activities meant that all experiences from each informant were used and developed in group discussions, which contributed to multifaceted and rich data. By including a second part of the workshop, focusing on how to overcome obstacles and reinforce the possibilities identified in the first part of the workshop, we were able to further use the informants’ thoughts and experiences.

Owing to the COVID-19 pandemic, the workshops were performed using the web-based video communication tool, Microsoft Teams. Although conducting qualitative studies over the internet facilitated the informants’ participation, it also involved some challenges [45]. A challenge was the informants’ familiarity with technological hardware and software. In this case, the informants had access to a reliable internet connection and a computer with a microphone and camera. The video communication tool, Microsoft Teams, was chosen because the informants were already familiar with this program because it was used in their organizations. All the informants (9/9, 100%) used digital communication tools in their daily work and were familiar with how to communicate using them. Another challenge is that web-based interviews seem to generate short responses and less contextual information [45], which could also apply to web-based workshops. Compared with in-person workshops, web-based groups need to be small, and 4 to 6 participants are recommended [45]. Therefore, we included a maximum of 6 informants in each workshop group. It might be a limitation that the informants in the workshops knew each other. A power imbalance might have occurred between managers and employees, which might have limited the issues that were raised for discussion during the workshops. However, the opinion of the 2 researchers participating in the workshops was that the informants spoke freely regarding the barriers to and facilitators of implementing the ICT tool. The fact that the implementation of the ICT tool was a project might have stimulated the informants to speak more freely than they might have done if the ICT tool was implemented as compulsory working task.

An exploratory design was considered appropriate for conducting research in this relatively underexplored field and thus, we adopted an inductive approach [30]. The choice of design was made to stimulate an open and creative discussion to enable the identification of new areas of determinants. In hindsight, a deductive approach would also have been possible to use because our results proved to be consistent with the checklist by Flottorp et al [26]. However, the use of the inductive approach contributed to a deep understanding of the determinants of implementing an ICT tool for social interaction among older people.

The findings present the determinants for the implementation of an ICT tool for social interaction among older people in municipal settings, but the determinants related to the ICT tool itself and older people are presented in a related article [22]. This division may complicate the possibility of obtaining an overall picture of the current determinants of importance for the implementation of the ICT tool. To make this easy for the reader, we refer to the related article by Gusdal et al [22] in the text.

The careful description of the data collection process and illustration of the findings with quotations increased the confirmability of the findings. Trustworthiness was strengthened through regular discussions among the authors during the analyses. The agreement between our findings and those of previous studies regarding the determinants of implementation in general [26] and implementation of welfare technology for older people in particular [25] increases the credibility of our findings, which is an important strength of our study and indicates wide transferability of the findings to the implementation of other ICT tools for older people in other contexts. However, the trustworthiness and transferability of the study results should be considered with caution because there were only 9 informants from 2 municipalities involved. Despite the small sample size, the study revealed important aspects to be considered when implementing ICT tools in municipality social services.

This study was conducted during the COVID-19 pandemic. The purpose was not to relate the results to the situation of older people specifically during the pandemic. However, the pandemic has probably affected the results in different ways. The problem of loneliness among older people increased during the COVID-19 pandemic [9-12], which increased the need for new solutions for communication among older people. This may have affected the attitude of both the older people and the personnel participating in this study toward ICT tools. The difficulties with meeting older people in person are also strongly associated with the restrictions during the pandemic and might have affected the results as the personnel did not have the prerequisites to meet older people and introduce the ICT tool (and the hardware) in the same way as without the pandemic and physical restrictions. Therefore, determinants with a direct connection to the pandemic are not as relevant during periods without a pandemic.

### Conclusions

The ICT tool discussed in this study will be used by older people in their homes for the purpose of social interaction. Although the ICT tool is for use by older people in their homes, with limited involvement of others, the results show that the implementation involves an entire organization at different levels. Specifically, the following may be required: ability of the personnel to introduce and support the ICT tool, including their competencies in using digital equipment; structure for communication within an organization; leadership as a driving force; sufficient resources; and possibilities to fulfill legal responsibilities. Regardless of how much each level within the organization comes into direct contact with the ICT tool, all levels need to be involved to create the necessary conditions for successful implementation. The prerequisites for the implementation of an ICT tool will probably change depending on the digital maturity of future generations. As this study only included 9 informants, the results should be considered with caution. The study was performed during the COVID-19 pandemic, which has probably affected the results.

## Appendix

Multimedia Appendix 1 Workshop guide.

## Abbreviations

EMA: effect modifier assessment
ICT: information and communication technology

## Data Availability

The data sets generated and analyzed during this study are available from the corresponding author upon reasonable request.
