# Supplementary material for: Determinants of Implementing an Information and Communication Technology Tool for Social Interaction Among Older People: Qualitative Content Analysis of Social Services Personnel Perspectives
Source: JMIR Aging. 2024 Feb 26;7:e43999. doi: 10.2196/43999 (PMC10928522; doi:10.2196/43999)
Supplement: Multimedia Appendix 1 [file aging_v7i1e43999_app1.pdf]

Multimedia Appendix 1. Workshop Guide.

This is a Multimedia Appendix to a full manuscript published in the J Med Internet Res Aging. For full copyright and citation information see http://dx.doi.org/10.2196/jmir.43999.

Workshop part 1

The following information regarding purposes of the study and the workshops are presented:

You have been invited to this evaluation of the Fik@ room since you all, in different ways, have experiences in introducing the Fik@ room to older people. This evaluation will be helpful for future implementation of the Fik@ room or other similar health and welfare technology.

Workshop part 1 questions/activities

1. **Let's start with a round of presentations where you can present yourself by name and in what way you have been engaged in introducing the Fik@ room to older people.**

Now it is time for you to take a pen and paper.

2. **I want you to think about what worked well when you introduced the Fik@ room to older people. What do you think made it work so well?**

Write down what worked well on sticky-notes, put only one thing on each sticky-note. This will be for your memory, and you are going to tell what you have written to the rest of us.

(Paus while everybody writes on their sticky-notes).

- How many sticky-notes did you write?
- Who wants to start talking about one of your sticky-notes?
- Does someone else have a sticky-note that reminds about this sticky-note? Please, tell us about your experiences.
- Is this something others of you recognize?

3. **I want you to think of what was difficult when you introduced the Fik@ room to older people. What do you think contributed to those difficulties?**

Write down what was difficult on sticky-notes, put only one thing on each sticky-note. This will be for your memory, and you are going to tell what you have written to the rest of us.

(Paus while everybody writes on their sticky-notes).

- How many sticky-notes did you write?
- Who wants to start talking about one of your sticky-notes?
- Does someone else have a sticky-note that reminds about this sticky-note? Please, tell us about your experiences.
- Is this something others of you recognize?

4. **Further questions (if not brought up during the workshop):**

- What did you think of the Fik@ room when you first got in contact with it? Have your thoughts about the Fik@ room changed during this period?
- How is the talking about the Fik@ room at your workplace?
- What is your perception about how the Fik@ room was received by the users?
- Did you encounter any problems with the technology and how could you handle the questions regarding this?
- Was time an issue? (If time aspects are not brought up)

Workshop part 2

Before workshop 2 data will be analyzed to formulate barriers and facilitators.

Workshop part 2 can be conducted directly after workshop 1 if the time of workshop 1 is not used.

Workshop part 2 questions/activities

The questions will be formulated so that the workshop participants can come up with solutions.

1. **How can we overcome the barriers?** (Identified barriers from workshop part 1 are worked through one by one)
2. **How can we strengthen what worked well?** (Identified facilitators from workshop part 1 are worked through one by one)

3. **Further questions (if not brought up during the workshop):**

- Are there any technology-coaches? Do you know of them? Do you think they could be helpful?
- Is it worth it to implement the Fik@ room? From whose perspective?
- Is there a need for the Fik@ room in your municipality? What do you think?
- Is there a need for this kind of product in your municipality? Are there better alternatives?

Procedure:

First workshop leader (JF) moderates the workshops.

Second workshop leader (CE) askes clarifying questions during the workshop and creates PowerPoints based on the participants sticky-notes.
